# Effects of Elevated Downstream Pressure and the Role of Smooth Muscle Cell Coupling through Connexin45 on Lymphatic Pacemaking

**DOI:** 10.3390/biom10101424

**Published:** 2020-10-08

**Authors:** Jorge A. Castorena-Gonzalez, Min Li, Michael J. Davis

**Affiliations:** 1 Department of Medical Pharmacology and Physiology, School of Medicine, University of Missouri, Columbia, MO 65212, USA; jcastorena@tulane.edu (J.A.C.-G.); lim@health.missouri.edu (M.L.); 2 Department of Pharmacology, School of Medicine, Tulane University, New Orleans, LA 70112, USA

**Keywords:** lymphatic vessel, connexin45, connexin43, connexin37, connexin47, lymphatic pacemaking, lymphedema

## Abstract

Lymphatic vessels rely on spontaneous lymphatic muscle cell (LMC) contractions and one-way intraluminal valves to efficiently pump lymph and return it into the bloodstream. Intraluminal pressure is known to regulate the contractile function of lymphatics, with pressure elevation leading to increased contraction frequency and decreased amplitude. Contractions are normally initiated by a dominant pacemaker and are highly entrained among strongly coupled LMCs. Previously, we found that connexin45 is the major connexin isoform mediating LMC-LMC electrical coupling. Lymphatics from mice lacking smooth muscle connexin45 display uncoordinated, impaired contractions. Here, we utilized this connexin45-deficient model, pressure myography, and recently developed, novel analytical tools to assess the effects of elevated downstream pressure on the number, location, and frequency of lymphatic pacemakers. Our results show that, in vessels from healthy controls, an increase in downstream pressure resulted in the recruitment/development of new pacemakers and increased contractile frequency while a dominant pacemaker continued to be observed. In contrast, vessels from connexin45-deficient mice displayed significantly more pacemakers, but none were dominant; this worsened with elevated downstream pressure. These results suggest a potential protective mechanism through which the lymphatic vasculature adapts to transient increases in downstream pressure, but which may not be sustained in scenarios with chronic elevated downstream pressure.

## 1. Introduction

It is now appreciated that Starling forces are not normally in balance across most blood microvascular networks, resulting in net fluid filtration and protein leakage into the interstitium that must be corrected by the lymphatic system [1]. After fluid and protein are taken up by the lymphatic capillaries, subsequent lymph transport depends in part on extrinsic forces (e.g., skeletal muscle contractions) and in part on the active contractions of collecting lymphatic vessels [2]. Therefore, critical to efficient lymph transport are the intrinsic spontaneous contraction of lymphatic muscle cells (LMCs), in combination with one-way lymphatic valves that prevent or retard lymph backflow [3,4,5].

In healthy collecting lymphatics, the contractions of all LMCs within a lymphangion (the segment between consecutive valves) [6], and even across multiple lymphangions, are highly coordinated, resulting in a contraction wave that conducts at 8–10 mm/s [7,8,9,10]. The entrainment of LMC contraction waves occurs as a result of the rapid propagation of a pacemaking signal (i.e., action potential) from LMC to LMC through connexin (Cx) gap junctions along the lymphatic wall. We recently demonstrated that strong electrical coupling between LMCs, with minimal interaction of other surrounding cell networks (e.g., limited electrical coupling between LMCs and lymphatic endothelial cells (LECs)), is essential for the focal generation of pacemaking signals and their rapid and efficient propagation along the lymphatic wall. Furthermore, the entrainment of lymphatic contractions is mediated primarily by LMC-LMC electrical communication through Cx45 gap junctions [7].

Along a network of actively pumping collecting lymphatic vessels, the cumulative effect of spontaneous contractions is a progressive increase in intraluminal pressure from the lymphatic capillaries to the first lymph node, with pressures becoming more pulsatile in the proximal regions of the network [11]. As a result, lymphatic collectors must normally transport lymph against an adverse hydrostatic pressure gradient. Additionally, this gradient will be exacerbated by gravitational loads, as it is in the blood vasculature [12]. Lymphatic contractions are known to be highly sensitive to changes in intraluminal pressure, which can modulate both contraction amplitude and frequency [13,14,15]. We previously demonstrated that selective elevation of downstream pressure had an effect on vessel tone and contractile frequency [16,17] even when the elevated pressure was experienced only by a small segment of the vessel on the downstream side of a closed lymphatic valve. These studies and others [8] suggest that the pacemaking initiation site is at least in part determined by the local pressure and/or wall tension. However, the influence of pressure on the location of the pacemaking initiation site and the degree of contraction wave entrainment has not been studied systematically.

## 2. Materials and Methods

### 2.1. Study Approval

All experimental protocols and procedures using animals were performed at the University of Missouri, these were all approved by the University of Missouri Animal Care and Use Committee and conformed to the US Public Health Service policy for the humane care and use of laboratory animals (PHS Policy, 2011).

### 2.2. Mice and Tamoxifen-Dependent Cre-Recombinase

C57BL/6J (WT) and Smmhc-CreER^T2^ (B6.FVB-Tg(Myh11-cre/ERT2)1Soff/J) mice were purchased from The Jackson Laboratory (Stock No.: 019079), Cx45^fx/fx^ mice were obtained from Klaus Willecke, University of Bonn, Germany. Smmhc-CreER^T2^; Cx45^fx/fx^ mice were generated through in-house breeding. Cre-recombination in Smmhc-CreER^T2^;Cx45^fx/fx^ mice was induced via feeding of tamoxifen-containing chow (ENVIGO TD.130855) for one week (~40 mg/kg of body weight per day assuming a 20–25 g body weight and 3–4 g intake). The recombination efficiency of this Smmhc-CreER^T2^ in lymphatic cells was previously assessed by crossing it with the fluorescent reporter ROSA26^mT/mG^; in that case, we showed recombination in >95% of the LMCs [18]. We also assessed the recombination efficiency specifically in Smmhc-CreER^T2^;Cx45^fx/fx^, showing that Cx45 was successfully deleted from ~98% of the LMCs [7]. Cx45^fx/fx^ (no Cre) mice were also fed tamoxifen-containing chow for one week and used as one of the control groups; a second control group of WT mice were fed regular mouse chow. Smmhc-Cre;eGFP and Smmhc-Cre;ROSA26^mTmG^ mice, used for Reverse Transcription-Polymerase Chain Reaction (RT-PCR) on Fluorescence Activated Cell Sorting (FACS)-purified LMCs, were originally obtained from The Jackson Laboratory (Stock No.: 007742 and 007676 respectively) and subsequently bred in-house. Mice were housed in groups of maximum 5 mice per cage under a 12-hour light/dark cycle. Room temperature was maintained at 22–25 °C. Mice had access to food and water at all times. All mice in this study received a control, regular mouse chow (PicoLab RodentDiet 20 Cat. No. 355043), except during the period for induction of Cre-recombinase (select groups).

### 2.3. Solutions and Chemicals

Krebs buffer contained: 146.9 mM NaCl, 4.7 mM KCl, 2 mM CaCl_2_·2H_2_O, 1.2 mM MgSO_4_, 1.2 mM NaH_2_PO_4_·H_2_O, 3 mM NaHCO_3_, 1.5 mM Na-HEPES, and 5 mM D-glucose (pH = 7.4). An identical buffer was prepared with the addition of 0.5% bovine serum albumin (BSA). During cannulation, Krebs-BSA buffer was present both luminally and abluminally; however, during the experiment, the abluminal solution was constantly exchanged with plain Krebs buffer. All chemicals were obtained from Sigma-Aldrich (St. Louis, MO, USA), with the exception of BSA (US Biochemicals; Cleveland, OH, USA), MgSO_4_, and Na-HEPES (ThermoFisher Scientific; Pittsburgh, PA, USA).

### 2.4. In Vivo Recording of Contractions of Popliteal Lymphatic Vessels

A WT mouse was first anesthetized by intraperitoneal injection of Ketamine/Xylazine (0.1 mL/25 g) and placed in prone position on a heated tissue dissection/isolation pad. Then, 2 µL of a 2% (*w*/*v*) FITC (fluorescein isothiocyanate-dextran, SIGMA Cat. No. FD2000S) solution in sterile saline was then injected into the dermis of the dorsal aspect of one foot. FITC solution was usually taken up by the initial lymphatics and visible in the collecting lymphatics 1–4 minutes after injection. In some rare cases, if the FITC solution was not observed within the lymphatic vasculature after 5 minutes, gentle massage of the foot was performed. The popliteal afferent lymphatic vessels were then exposed by making a proximal-to-distal incision of the skin beginning at the ankle. BSA-containing Krebs solution (at room temperature) was constantly added to ensure the exposed tissue remained moist. The mouse was then transferred onto the stage of a Zeiss AXIO Zoom V16 Fluorescence Microscope and place in the prone position. The incised area of skin containing the exposed lymphatic vessel was then covered with a metal plate containing a 0.17 mm-thick glass window heated to 36 °C using a Warner Instruments TC-344B temperature controller. The exposed tissue underneath the window was constantly perfused with warm (37 °C) Krebs solution. Once the preparation was stable, fluorescence videos were acquired and the spontaneous contractions of a collecting lymphatic were acquired at 10–20 fps through a 1x Zeiss macroscope objective using a Hamamatsu C11440 ORCA-Flash4.0 digital camera (2048 × 2048 pixels) [7]. Custom-written Python-based programs were used to automatically detect changes in diameter over time from a representative window/region of interest. These diameter traces were then used to calculate the contractile and pacemaking parameters defined in Section 2.7.

### 2.5. Vessel Isolation, Pressure Myography, and Data Acquisition

Afferent popliteal lymphatic vessels were isolated as previously described [14,19,20,21,22]. Briefly, a mouse was anesthetized by intraperitoneal injection of Ketamine/Xylazine (0.1 mL/25 g) and placed on a heated pad, in the prone position, for tissue dissection. Loss of pedal and pinna reflexes was assessed prior to and during dissection to ensure adequate level of anesthesia. The area surrounding the superficial saphenous vein was exposed by making an incision along the calf area and the afferent popliteal lymphatic vessels along both sides of the major vein were isolated. Each lymphatic vessel was pinned onto a dissection chamber (bottom coated with Sylgard) using 40 µm stainless steel wire. The dissection chamber contained a Krebs (BSA-containing) buffer at room temperature. During dissection, the large majority of the adipose and connective tissues surrounding each lymphatic vessel were cleared by microdissection. Lymphatic segments were studied in 3-mL observation chambers, where each segment was cannulated and pressurized using two glass micropipettes (50–60 µm O.D.) mounted on probe holders secured on micro-manipulators (MX10 Siskiyou Corporation). Lymphatic segments contained 1–3 valves. The pipette systems, with cannulated lymphatic vessel segment, were transferred onto the XY-stage of an inverted confocal microscope Olympus IX81 for observation. Polyethylene tubing was attached to the back of each glass micropipette and then connected to a 2-channel microfluidic pressure controller (Elveflow OB1 MK3, Paris) with attached low-pressure transducers. The vessel was then allowed to equilibrate at 37 °C for about 30 minutes (until frequency and amplitude of contractions stabilized) with both upstream and downstream pressures set to 3 cmH_2_O. Constant perfusion with Krebs buffer was maintained using a peristaltic pump at a rate of 0.5 mL/min. Custom-written LabVIEW programs (National Instruments; Austin, TX) acquired real-time video of the lymphatic preparation [23]. Videos of the contractile activity of lymphatic vessels were recorded for further analyses in brightfield mode at 30–250 fps using a Basler acA2000-340km camera.

### 2.6. Assessment of Contractile Function of Isolated Lymphatic Vessels

The contractile phenotype of each lymphatic vessel was characterized under control conditions, with upstream (P_upstream_) and downstream (P_downstream_) pressures set to 2 cmH_2_O, and then under an adverse differential pressure gradient (P_downstream_>P_upstream_) using various pressure combinations to impose different levels of differential pressure gradient ranging from 0.5 to 4.5 cmH_2_O. Some of these combinations included pressures set at (upstream-downstream in cmH_2_O): 2–3 and 2–5 as shown in Figure 5; others included: 0.5–2, 0.5–3, 0.5–5, 1–2, and 1–3. In all cases, lymphatic vessels had at least one intraluminal valve. Spontaneous contractions were then recorded, under no flow conditions, for either 5 or 15 minutes (depending on the experimental protocol) at each given pressure setting. The contractile function under control and differential pressures was then analyzed using two-dimensional Space Time Maps (STMs) of contractions (see Section 2.6) and the corresponding pacemaking activities were compared. Live-tracked diameter was detected using custom-written LabView programs and used to calculate various parameters that characterize the contractile and pacemaking function of lymphatic vessels (see Section 2.7).

### 2.7. Assessment of the Initiation and Propagation of Contraction Waves using Space Time Maps (STMs)

**Figure 1:**
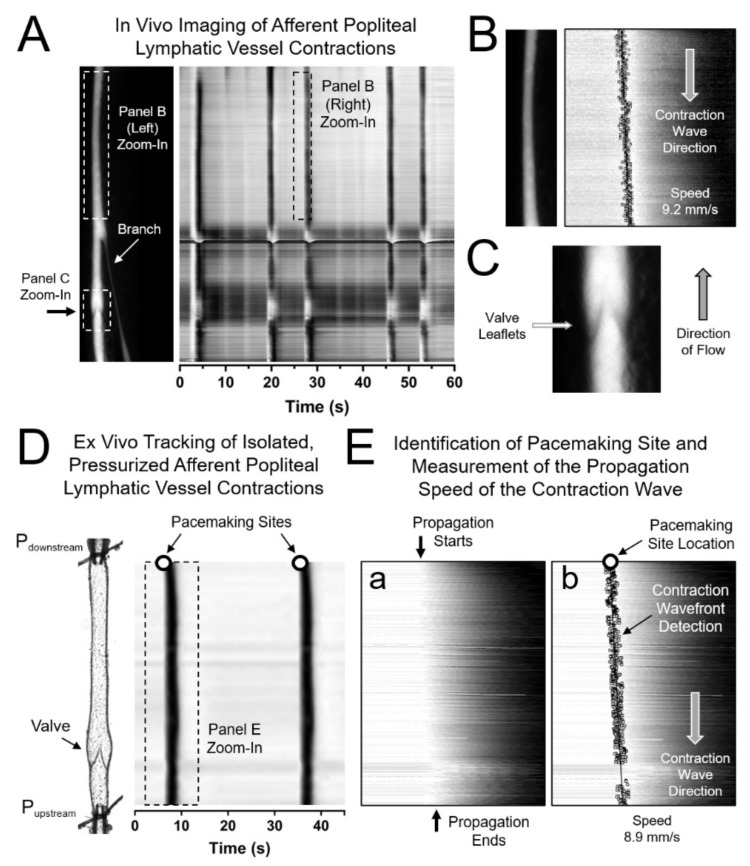
In vivo and ex vivo assessment of afferent popliteal lymphatic vessel contraction waves: (**A**) In vivo imaging of the lymphatic vasculature in the hindlimb of a mouse following an injection of FITC tracer into the upstream popliteal lymphatic network. Five contractions along the entire imaged lymphatic segment are represented in a two-dimensional map (Space Time Map—STM) as 5 dark vertical bands extending along the entire height of the image/map); (**B**) Analysis of the propagation of the contraction wavefront for each contraction using STMs allows assessment of the direction of propagation and measurement of propagation speed in vivo; (**C**) In vivo image of a lymphatic valve; (**D**) STMs of contractions from isolated, cannulated, pressurized afferent popliteal lymphatic vessels; (**E**) Similar to the analyses performed in vivo, automated analysis of the propagation of the contraction wavefront allowed us to determine where the contraction initiated (the pacemaking site) as well as the direction and speed of propagation. In panel E-a, notice the horizontal shift between where the contraction wavefront starts at the top of the image and where the contraction ends (bottom), which is right-shifted in the transformed image. Conduction/propagation speed is directly associated with the slope of the line that connects all the points along the propagation wavefront.

Fluorescence videos of contractions recorded in vivo (i.e., FITC-solution-filled lymphatics) and bright-field videos of contractions recorded ex vivo (i.e., pressure myography) were processed and analyzed to generate 2-dimensional maps (STMs) representing the measurement of the outside diameter (encoded in 8-bit grayscale) over time (horizontal axis) at every position along the vessel (vertical axis). To automatically measure the outside diameter over time along the entire vessel segment, videos of lymphatic spontaneous contractions, either in vivo or ex vivo, were processed frame by frame; each frame was dynamically thresholded and the lymphatic walls were then detected via contour detection. Edge detection of the identified walls allowed us to determine the distance between the outside edges of the lymphatic wall at each specific position along the longitudinal axis. In these maps, darker shades indicate a smaller diameter (i.e., systole), while lighter shades correspond to a larger diameter (i.e., diastole). Each vertical dark band thus indicated a single contraction, and bands that span the entire STM represent contractions that propagate through the entire vessel segment. In some STMs, horizontal band patterns were observed, these usually were artifacts caused by poor tracking associated with the lack of a clean wall edge or weaker contractions that typically occur at valve areas. The speed and direction of each propagating contraction wave (both ex vivo and in vivo) were computed from the STMs by automatically detecting the propagation of the contraction wavefront (Figure 1); the resulting array of data points was fit with a linear function and the slope, which was directly associated with the propagation/conduction speed, was obtained. Note that the STMs made from in vivo images contain more artifacts and irregularities than STMs from ex vivo vessels due to side branches, crossing over of blood vessels and tissue movement. All video processing and 2-dimensional analyses were performed using a set of custom-written Python-based programs [7]. It should be noted that this edge-detection for STM generation applies to a limited type of lymphatic collector (e.g., popliteal, superficial cervical) with rapid, relatively large-amplitude contractions [22]; lymphatic collectors from other regions of the mouse with small amplitude contractions (e.g., mesentery) or slower, sine-wave like contractions (e.g., inguinal-axillary) would need modified algorithms for application of this method.

### 2.8. Calculation of Contractile and Pacemaking Parameters

End diastolic diameter (EDD) and end systolic diameter (ESD) were extracted from outside diameter data recorded from contracting vessels in vivo and ex vivo. EDD and ESD were used to calculate contraction amplitude and ejection fraction. These contractile parameters are defined as follows:

(1) Contraction Amplitude = EDD−ESD

(2) $$Percent\;Conduction\;Length\;\left(PCL\right)\;=\;\left(\frac{L_{contraction}}{L_{vessel}}\right)*100$$

(3) $$Ejection\;Fraction\;=\;\left(\frac{PCL}{100}\right)\left[\frac{EDD^{2}-ESD^{2}}{EDD^{2}}\right]$$

where L_contraction_ is the length of the lymphatic segment that displayed an entrained, coordinated contraction, while L_vessel_ is the length of the entire lymphatic segment (in general, these two are identical in vessels from control, healthy humans and animals, e.g., mice, rats, etc.).

In order to assess the pacemaking function of lymphatic vessels ex vivo and characterize the activity of different pacemaking sites, the following parameters were calculated:

(4) $$Switching\;Probability\;\left(P_{sw}\right)\;=\;\left(\frac{N_{c}}{N_{sw}}\right)*100$$

where N_c_ is the total number of contractions and N_sw_ is the total switching count, a count is defined as a contraction that initiated from a different pacemaker compared to the immediate previous contraction. Here, we assessed the pacemaking activity of lymphatic vessels when downstream pressure was differentially elevated. Under an adverse pressure gradient, one of the intraluminal valves within the lymphatic segment closed, preventing backflow. Ideally, this valve would be located exactly in the middle point along the segment (i.e., equal distance to the upstream and downstream pipettes); however, this is usually not the case. In order to account for this deviation from the middle position, a normalized switching probability was calculated as follows:

(5) $$Normalized\;P_{sw}\;=\;\left\{\begin{array}{c} P_{sw}*\left(1+DL_{diff}\right),\;DL_{diff}\leq 0\\ P_{sw}+\left[\left(100-P_{sw}\right)*DL_{diff})\right],\;DL_{diff}>0 \end{array}\right.$$

where DL_diff_ is the normalized difference, from the middle point (i.e., 0.5), in the length of the downstream section of the lymphatic segment (L_downstream_), calculated as:

(6) $$DL_{diff}\;=\;0.5-\left(\frac{L_{downstream}}{L_{vessel}}\right)$$

### 2.9. Fluorescence Activated Cell Sorting (FACS) and End-point RT-PCR

Populations of LMCs were sorted and purified from mouse lymphatic vessels by means of FACS as previously described [24]. Briefly, intact inguinal-axillary lymphatic vessels were isolated [22] from Smmhc-Cre;eGFP or Smmhc-Cre;ROSA26^mTmG^ mice and cleaned of adipose and connective tissue. Vessels were then enzymatically digested using cocktails containing collagenase H and F, papain, trypsin inhibitors, and elastase. The resultant dispersed cells were then sedimented by centrifugation at 300 g for 4 minutes. Cells were washed, resuspended, and filtered (through a 35-µm nylon filter) to obtain single cells in suspension (0.6 mL final volume). Myh11+ cells expressing eGFP (i.e., LMCs) were FACS-sorted with a Beckman-Coulter MoFlo XDP instrument (Cell and Immunobiology Core Facility, University of Missouri) and recollected into lysis buffer. Subsequently, total RNA was extracted from sorted cells using the Arcturus PicoPure Isolation kit. Purified RNA was then used for cDNA synthesis via reverse transcription. mRNA expression was assessed by RT-PCR. All primers were designed to amplify intron-spanning RNA regions. Primer sequences used in the experiments are listed in Table 1.

### 2.10. Statistical Analysis

The number *n* refers to the total number of animals included per group. In most cases, multiple lymphatic segments were studied from the same animal, in which case the results from those vessels were averaged and consolidated into a single *n*-th entry. Statistical differences in the various contractile function parameters were assessed via paired or unpaired t-tests (parametric) when comparing only 2 groups and one-way ANOVA with correction for multiple-comparisons using either Dunnett’s test (when the data from two or more groups was compared to a control data set) or Tukey’s test (when the data from more than two groups was compared with every other group). All statistical analyses were performed using GraphPad Prism 8. Results are reported as mean ± SEM with significance set at *p* < 0.05.

## 3. Results

### 3.1. In Vivo and Ex Vivo Assessement of the Initiation Sites of Spontaneous Contractions and Measurement of the Direction and Speed of the Associated Propagating Contraction Wave in Lymphatic Vessels

We first assessed the sites for the initiation of contraction waves in lymphatic vessels from control (C57BL/6J) mice in vivo. Following a 2-µL injection of 2% FITC (*w*/*v* in sterile saline) in the dorsal surface of the foot, the fluorescent tracer was allowed to be taken up by the lymphatic capillaries. Once transport of the fluorescent tracer was visible in the collecting lymphatic network (Figure 1A left panel), the preparation and contraction pattern were allowed to stabilize at 37 °C for 10 minutes and then fluorescence videos of contraction waves in afferent popliteal lymphatic vessels were recorded (see Methods section for a detailed description). These videos were then processed and analyzed to construct two-dimensional Space Time Maps (STMs) that represented the local change in outside diameter as a function of time at each position along an entire lymphatic segment (Figure 1A right panel). As previously described [7], analysis of the contraction band pattern allowed determination of the direction and speed of the propagating contraction wave (Figure 1B). Direction of flow was determined from the orientation of the intraluminal lymphatic valves (Figure 1C). In the in vivo scenario, assessment of the direction of the contraction wave allowed us to determine whether a given contraction initiated upstream (toward the capillaries) or downstream (near the popliteal lymph node) from the observation site; however, identification of the exact location of the pacemaking/initiation site was not possible as in most cases contractions initiated outside the field of view. A correlation between local intraluminal pressure and pacemaking activity could not be established as intraluminal pressures are unknown in vivo. In contrast, ex vivo recordings of the spontaneous contractions of isolated lymphatic vessels, using pressure myography, allowed assessment of their contractile function under controlled intraluminal pressure and flow (i.e., no imposed flow) conditions (Figure 1D). In addition, STM analysis of contractions (generated from recorded brightfield videos of isolated contracting lymphatic segments) also allowed determination of the exact location (with an approximate precision of ≤60 µm) of the pacemaking initiation site (Figure 1D,E).

### 3.2. Lymphatic Contractions Display a Higher Probability of Initiating Downstream in the Lymphatic Network

**Figure 2:**
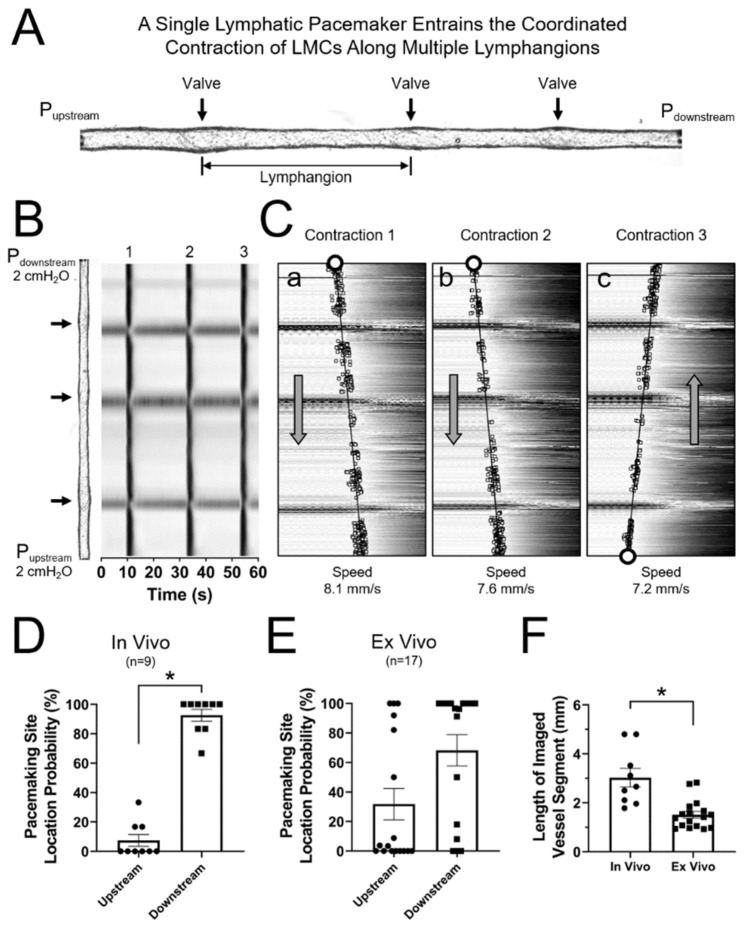
Assessment of the location of the initiation sites for contractions (i.e., pacemaking sites) in ex vivo preparations of popliteal lymphatic vessels: (**A**) A long isolated, cannulated, pressurized lymphatic segment containing 3 valves; (**B**) Three consecutive spontaneous contractions recorded from the lymphatic segment shown in panel A under equal upstream and downstream pressures (2 cmH_2_O); the image of the vessel is rotated 90^o^ and aligns with the STM. (**C**) Analysis of each individual contraction wave shows two contractions being initiated on the downstream (top) end of the lymphatic segment, while the last contraction originates from a pacemaking site located in the opposite (i.e., upstream) end. Pacemaking sites are represented by open markers (circles). Contraction waves associated with different pacemaking sites display comparable propagation speeds; (**D**,**E**) Pacemaking location probability in vivo and ex vivo respectively; (**F**) Length of lymphatic segments included in this analysis. * Indicates statistical significance (*p <* 0.05) between groups.

Once initiated, lymphatic contractions could entrain the contraction of all lymphatic muscle cells (LMCs) within a lymphangion (the segment between consecutive valves) and even across multiple lymphangions. In the example shown in Figure 2A,B, an afferent popliteal lymphatic vessel pressurized to 2 cmH_2_O (no flow, P_upstream_ = P_downstream_), containing 3 valves, 2 full lymphangions, 2 partial lymphangions, and ~2.9 mm in length, exhibited entrained contractions that initiated at one end of the vessel preparation (i.e., near one of the cannulating pipettes) and then propagated at speeds of 7–8 mm/s along the entire segment. Note that the first two contractions initiated at the downstream end of the vessel (Figure 2C panels a,b), while the third contraction initiated at the upstream end (Figure 2C panel c). Under these specific conditions, there were at least 2 active pacemaking/initiation sites in this particular vessel. Therefore, we asked the question: What is the probability that a contraction will be initiated at the upstream or at the downstream end? To answer this question, we recorded and analyzed every contraction of the afferent popliteal lymphatic vessels both in vivo (5-minute recordings) and ex vivo (15-minute recordings). Ex vivo contractions were recorded from vessels pressurized to 2 cmH_2_O under no-flow conditions. Interestingly, we found that in vivo lymphatic contractions had a significantly higher probability of being initiated downstream, with a mean probability (±SEM) of 92.6±4.0% (*p <* 0.05). The ex vivo preparation showed a similar, but not statistically significant, trend with a mean probability (±SEM) of 68.2 ± 10.6% (Figure 2D,E). In all cases, contractions propagated throughout the entire length of the lymphatic segment under study. In the in vivo preparation, the total length analyzed was limited by the field of view and the length of the segment in focus; while in the ex-vivo preparation, the segment length for analysis is determined by the section of a lymphatic vessel that did not contain branches (as pressure needs to be maintained constant) and by the expertise/skill of the person dissecting and cleaning the vessel, to ensure no damage. The average lengths of the lymphatic segments analyzed are shown in Figure 2F.

### 3.3. Efficient Entrainment of Lymphatic Contractions Requires a Dominant Pacemaker and Strong Electrical Coupling between LMCs through Connexin 45-Containing Gap Junctions

**Figure 3:**
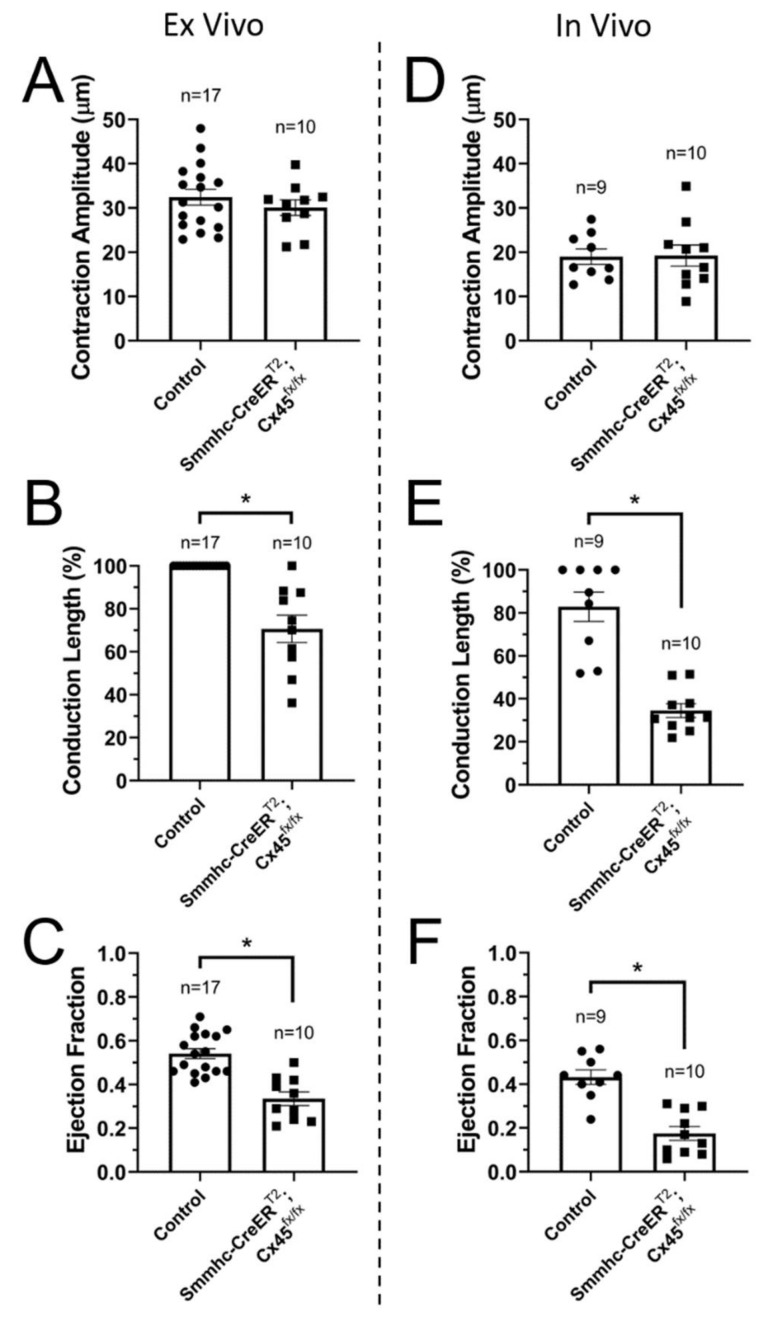
Ex vivo and in vivo assessment of the contractile function of lymphatic vessels from control (C57BL/6J WT and Cx45^fx/fx^ combined) and smooth-muscle Cx45-deficient (Smmhc-CreER^T2^;Cx45^fx/fx^) mice: (**A**,**D**) Contraction amplitude; (**B**,**E**) Conduction length; and (**C**,**F**) Ejection fraction for ex vivo and in vivo protocols respectively. Ex vivo, contractions were recorded under 2 cmH_2_O of intraluminal pressure (P_upstream_ = P_downstream_). * Indicates statistical significance (*p <* 0.05) between control and SM-Cx45-deficient groups.

Isolated, pressurized lymphatic vessels (ex vivo) from control mice display contractions that are initiated by at most two pacemaking sites, with one pacemaker usually acting as the dominant one. These contractions are mostly initiated at the ends/edges of the preparation (i.e., close to where the vessel is tied to the cannulating pipette). This may have to do with the fact that a pacemaking signal originating in an LMC located at one edge of the preparation may be intrinsically stronger, as it has only one option for propagation (current sink), i.e., towards the rest of the lymphatic segment, as opposed to when it originates in an LMC in the middle of the lymphatic segment, where the pacemaking signal strength will be split by bidirectional current sinks. This also makes LMCs located at or near valve areas, where the density of LMCs is lower, and presumably the LMC-LMC coupling is weaker, more likely to become the pacemakers; we have previously demonstrated these principles using numerical models [25]. In order to assess the degree of dominance by the different pacemakers, we started by recording and characterizing the contractile function of afferent popliteal lymphatic vessels from control (C57BL/6J WT and Cx45^fx/fx^) and smooth-muscle (SM) Cx45-deficient (Smmhc-CreER^T2^;Cx45^fx/fx^) mice under control conditions (i.e., no flow, equal upstream and downstream pressures), pressurized to 2 cmH_2_O over a 15-minute period (~60–170 recorded contractions per vessel). Contraction amplitude, conduction length, and ejection fraction were calculated (Figure 3A–C). Our results confirm what we have previously reported [7], that deletion of SM-Cx45 results in impaired lymphatic contractions with significantly decreased ejection fraction, as a result of the lack of entrainment of LMC-contraction (i.e., reduced percent conduction length) due to limited electrical coupling between LMCs. However, local contraction amplitude, as recorded from a representative window of interest, was not significantly affected by the deletion of SM-Cx45. Similar results and observations were confirmed for contractions recorded in vivo (Figure 3D–F).

**Figure 4:**
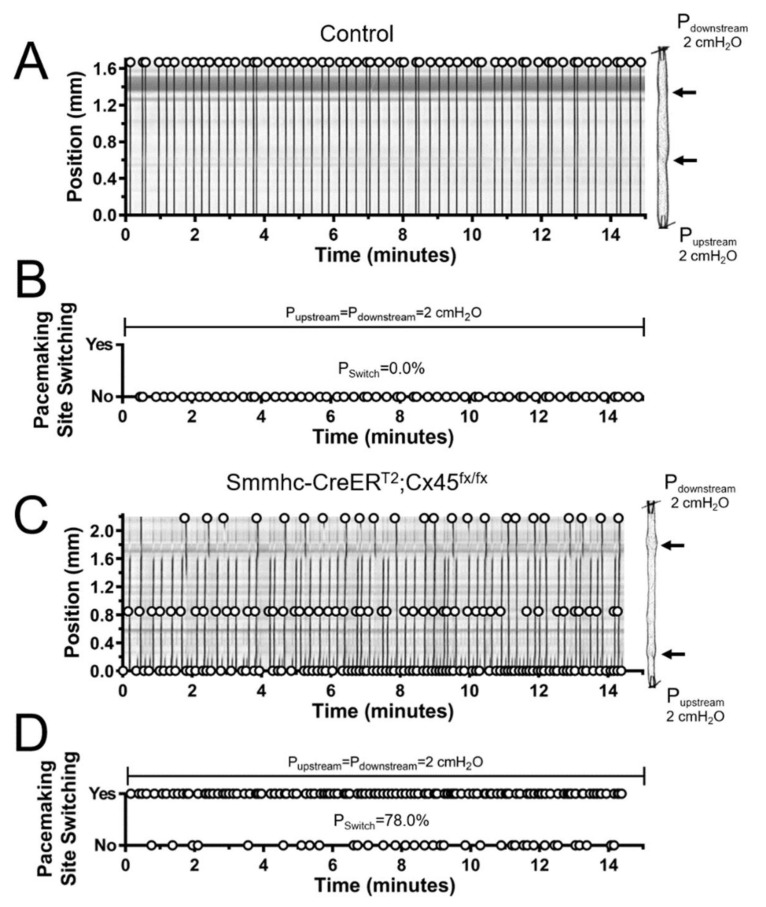
Pacemaking activity in lymphatic vessels from control (C57BL/6J WT and Cx45^fx/fx^) and smooth-muscle Cx45-deficient (Smmhc-CreER^T2^;Cx45^fx/fx^) mice under control intraluminal pressure (P_upstream_ = P_downstream_ = 2 cmH_2_O): (**A**,**C**) STMs of contractions of popliteal lymphatic vessels recorded over 15 minutes from control and Cx45-deficient animals. Vessel images are aligned to the right sides of the two-dimensional maps; here, valves are indicated with dark arrows. Pacemaking sites are represented by open circles. (**B**,**D**) Pacemaking site switching determined after comparing where consecutive contractions were initiated. Comparison is performed one-by-one for all contractions in the STMs shown in A and C. Switching probability (P_Switch_) is displayed for each given example.

**Figure 5:**
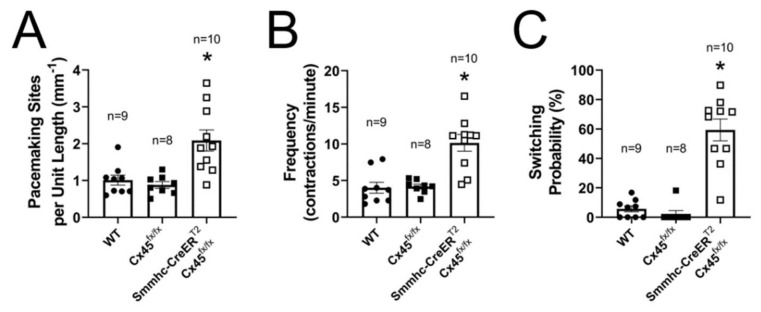
Summary analysis of the pacemaking activity in lymphatic vessels from control (C57BL/6J WT and Cx45^fx/fx^) and LMC Cx45-deficient (Smmhc-CreER^T2^;Cx45^fx/fx^) mice under control intraluminal pressure (P_upstream_ = P_downstream_ = 2 cmH_2_O): (**A**) Number of pacemaking sites per unit length; (**B**) Pacemaking frequency (frequency of contractions); and (**C**) Calculated pacemaking switching probability. * Indicates statistical significance (*p <* 0.05) when compared to all other unmarked groups.

Subsequently, we analyzed these recordings of the contractile activity of lymphatics and determined where each contraction was initiated. To test the hypothesis that the development of a single, dominant pacemaker, which is likely necessary for the efficient entrainment of lymphatic contractions, requires strong electrical coupling between LMCs through gap junctions, we compared our results for contractions of vessels from control (C57BL/6J or tamoxifen-treated Cx45^fx/fx^) and SM-specific Cx45-deficient mice (Figure 4). We showed previously that recombination occurs in >98% of popliteal LMCs with the Smmhc-CreER^T2^ [7,18]. The example experiment in Figure 4A shows an STM of the contractile activity of a control vessel with time on the horizontal axis. As indicated by the markers (open-circles), each contraction (dark vertical bands) was initiated by the same pacemaker (position—vertical axis, measured from the upstream end) which entrained the coordinated contraction of all LMCs in the entire segment. Therefore, comparisons of consecutive contractions revealed no switching of the pacemaking site, as shown in Figure 4B. In contrast, contraction waves in lymphatic vessels from SM-Cx45-deficient animals were initiated by multiple pacemakers, and in most cases, these pacemaking signals failed to entrain the contraction of all LMCs in the entire lymphatic segment (as indicated by cut-off dark vertical bands) (Figure 4C). These pacemaking sites constantly alternated, as indicated by the higher frequency of pacemaking site switching shown in Figure 4D. We calculated the *Switching Probability* (P_switch_), defined as the percent chance that consecutive contractions will be initiated by a different pacemaking site. Therefore, P_switch_ = 0% indicates that all contractions originated from a single dominant pacemaking site (as it is the case for the control example in Figure 4A,B) and P_switch_ > 0% indicates contractions were initiated by at least 2 different pacemaking sites. Other notable values are P_switch_ < 50% (and ≠0) indicates at least 2 pacemaking sites were observed with one being dominant, and P_switch_ > 50% indicates at least 2 pacemaking sites were observed but none were dominant (as is the case for the example shown in Figure 4C,D). Consistent with what we have previously found [7], Cx45-deficient vessels have significantly more pacemaking sites per unit length (Figure 5A) due to impaired electrical coupling between LMCs. Calculation of the switching probability showed that most contractions in control vessels were initiated by a single, dominant pacemaker, whereas lack of LMC-Cx45 resulted in the loss of a dominant pacemaker, as indicated by a high switching probability (P_switch_ > 50%) (Figure 5C). Altogether, the loss of a dominant pacemaker and the significant increase in number of pacemaking sites per unit length in vessels from Cx45-deficient mice resulted in an overall higher contraction frequency (Figure 5B).

**Figure 6:**
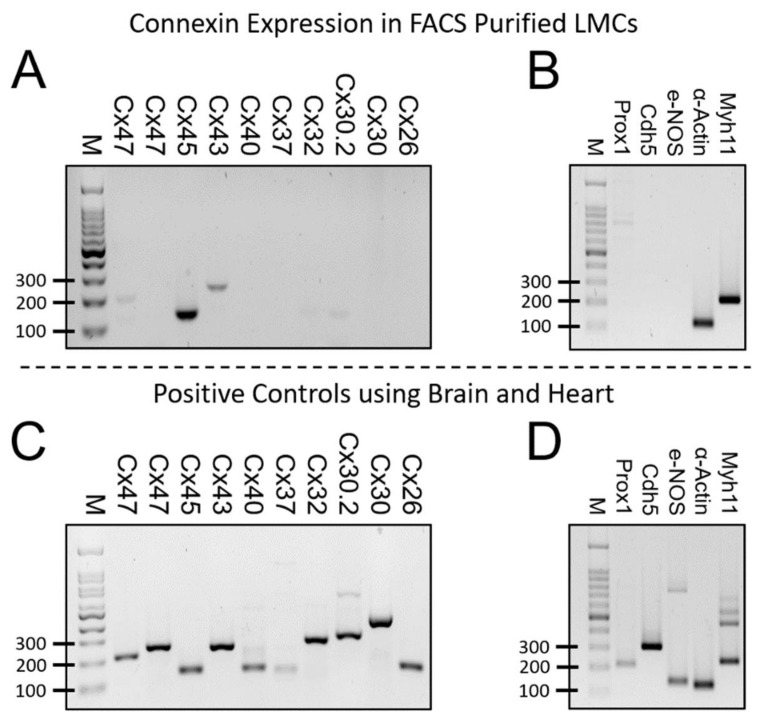
Connexin isoform mRNA expression (RT-PCR) on FACS purified LMCs from control mice: (**A**) mRNA expression for various Cx isoforms in FACS-purified LMCs, showing expression of Cx45 and to a lesser extent Cx43 and Cx47; (**B**) Assessment of LMC purity by determining mRNA expression of markers commonly observed in LMCs and LECs. Purified LMCs show strong α-actin and Myh11 message, while the LEC markers Prox1, Cdh5, and e-NOS are undetectable; and positive control tests using (**C**) brain or (**D**) heart tissues, for the gene targets shown in panels A and B. Representative of 5 gels on 5 different samples/animals.

As shown here, and reported in our previous study [7], Cx45 is critical in mediating the electrical coupling between LMCs; however, an interesting observation is that lymphatic contractions in SM-Cx45 deficient mice, although they display significant lack of LMC-contraction entrainment, appear to retain some limited LMC-LMC coupling. This is evident by slower and shorter propagating contraction waves (i.e., decreased conduction length) that result from the entrained contraction of groups of fewer LMCs, as opposed to all LMCs, in a given segment, being entrained by a single pacemaking signal in lymphatics from control mice. Therefore, a question that arises is: What mechanism(s) are responsible for this residual LMC-LMC coupling in SM-Cx45 deficient lymphatic vessels? Possible answers to this question are: 1) other connexin isoforms may normally be present in LMCs; and/or 2) deletion of Cx45 may lead to the compensatory upregulation of other connexin isoforms. Using RT-PCR, we previously reported that lymphatic vessels (whole vessel samples) from humans and mice consistently show mRNA expression for five connexin isoforms: Cx37, Cx40, Cx43, Cx45, and Cx47; mRNA for other isoforms is sometimes detected at very low levels [7]. Using methods previously described, we utilized fluorescence activated cell sorting (FACS) to obtain purified populations of LMCs from lymphatic vessels freshly isolated from Smmhc-Cre;eGFP or Smmhc-Cre;ROSA26^mTmG^ mice, which express the green fluorescence protein eGFP in Myh11+ cells (i.e., in smooth muscle cells). In purified LMCs, we found a prominent band for Cx45, a weaker band for Cx43, and a very weak band for Cx47 (Figure 6A). To assess the purity of the sorted LMC populations, we determined the expression of common LMC or LEC markers Myh11, α-smooth muscle actin, Prox1, Cdh5, and e-NOS. No mRNA expression was detected for the LEC markers Prox1, Cdh5, or e-NOS, while strong mRNA signal was observed for the SM-markers Myh11 and α-actin, see Figure 6B. Brain or heart tissues were used as positive controls for the primers used in these experiments (as shown in Figure 6C,D). The absence of LEC contamination is important because LECs highly express Cx43 and Cx37 [26].

### 3.4. Lymphatic Pacemaking can be Regulated by Localized Changes in Intraluminal Pressure

**Figure 7:**
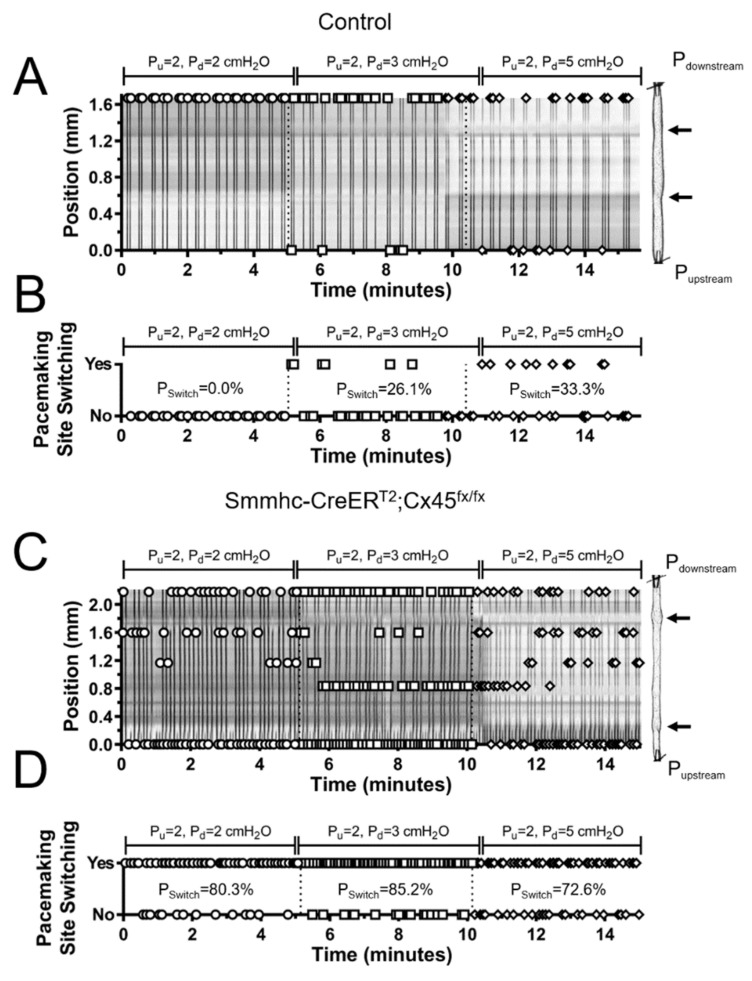
Pacemaking activity in lymphatic vessels from control (C57BL/6J WT and Cx45^fx/fx^) and LMC Cx45-deficient (Smmhc-CreER^T2^; Cx45^fx/fx^) mice under differentially increased downstream (downstream) intraluminal pressure (P_downstream_>P_upstream_): (**A**,**C**) STMs of contractions of popliteal lymphatic vessels recorded over 15 minutes from control and Cx45-deficient animals. Different combinations of various upstream–downstream pressure levels (denoted as P_u_ and P_d_ respectively) were tested over periods of 5 minutes each. Vessel images are aligned to the right sides of the two-dimensional maps (dark arrows indicate valve location). Pacemaking sites are represented by open circles; (**B**,**D**) Pacemaking site switching determined after comparing where consecutive contractions were initiated. Comparison is performed one-by-one for all contractions in the STMs shown in A and C. Switching probability (P_Switch_) is displayed for each given example and for each given differential pressure level.

**Figure 8:**
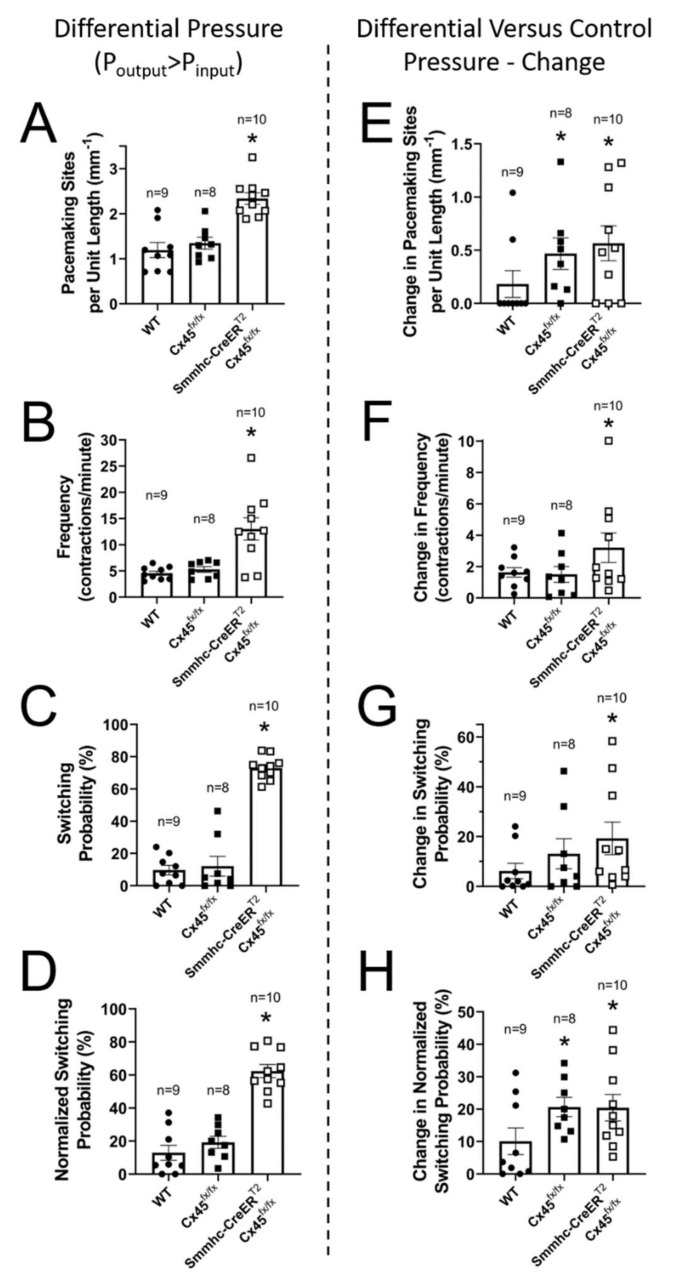
Summary analysis of the pacemaking activity in lymphatic vessels from control (C57BL/6J WT and Cx45^fx/fx^) and LMC Cx45-deficient (Smmhc-CreER^T2^;Cx45^fx/fx^) mice under differentially increased downstream intraluminal pressure (P_downstream_ > P_upstream_): (**A**) Number of pacemaking sites per unit length; (**B**) Pacemaking frequency (frequency of contractions); (**C**) Calculated switching probability; (**D**) Calculated normalized switching probability; (**E**–**H**) Change in number of pacemaking sites per unit length, change in frequency, change in percent switching probability, and change in normalized switching probability for pacemaking parameters calculated as the difference in pacemaking parameters measured under differential pressure conditions versus control pressures (equal upstream and downstream pressures set at 2 cmH_2_O). * Indicates statistical significance (*p <* 0.05) when compared to all other unmarked groups.

We then tested the hypothesis that pacemaking can be regulated by localized changes in intraluminal pressure, specifically, an elevated downstream pressure. We recorded contraction waves under conditions of imposed differential pressure, using the same set of vessels included in the previous section (Figure 4; Figure 5), all of which had at least one valve. This allowed us to set different intraluminal pressures in different sections of a given lymphatic segment by independently controlling upstream and downstream pressures. The number of valves per lymphatic segment was similar between control and SM-Cx45-deficient vessels, i.e., 1.7 ± 0.2 and 1.9 ± 0.2, respectively. We tested a series of upstream–downstream pressure combinations that imposed different levels of differential pressure across these lymphatic segments and recorded contractions at each set of pressures for 5 minutes. Some of these combinations included pressures (upstream–downstream in cmH_2_O): 2–3 and 2–5 as shown in Figure 7; other tested combinations included 0.5–2, 0.5–3, 0.5–5, 1–2, and 1–3. It is important to note that, in all cases, downstream pressure was set higher than upstream pressure, forcing one of the valves in the lymphatic segment to close, preventing backflow. The mean differences in upstream and downstream pressures (i.e., pressure gradient across the valve) and the overall mean pressures along the entire lymphatic segment were 2.4 and 2.5 cmH_2_O, respectively. While we were not able to establish any clear correlation between changes in pacemaking activity and the specific degree/level of differential pressure (e.g., the higher the differential pressure, the higher the number of active pacemakers), we did observe that regardless of the upstream–downstream pressure combinations, differentially elevated downstream pressure induced a significant increase in pacemaking activity compared to the scenario where both upstream and downstream pressures were equal (i.e., 2 cmH_2_O). We observed an overall higher number of pacemaking sites, significantly increased switching probability, and increased contraction frequency (Figure 8A–C). A complicating factor in this analysis was the fact that the location of valves was different in each lymphatic segment so that the percent length of the vessel segment that experienced a higher pressure compared to lower pressure was different in each preparation. Ideally, for these experiments we would want a 50/50 upstream-length/downstream-length ratio, with 50% of the vessel segment under lower pressure and the remaining 50% set to a higher pressure. To account for the deviation from the ideal 50/50 ratio, we calculated a normalized switching probability (Figure 8D). Finally, to determine the specific effect of differentially increasing the intraluminal pressure in the downstream side of each lymphatic segment, we calculated the mean change in: 1) number of pacemaking sites, 2) frequency, 3) switching probability, and 4) normalized switching probability when comparing the pacemaking activity of lymphatic vessels under equal (2 cmH_2_O, Figure 5) pressure versus differential pressure (Figure 8A–D). These mean differences are shown in Figure 8E–H. Importantly, all pacemaking parameters showed a positive difference, suggesting that local changes in intraluminal pressure, in this case, a selective increase in upstream pressure, regulated lymphatic pacemaking. Specifically, elevated upstream pressure resulted in a higher number of pacemaking sites and increased overall contraction frequency as a result of more active pacemakers (especially those in the higher-pressure region).

## 4. Discussion

While lymph can be transiently propelled forward due to compression of the lymphatic vasculature during skeletal muscle contraction, under many conditions the spontaneous contractile activity of lymphatic vessels is required for lymph transport [27]. Lymphatic contractions are driven by electrical signals (i.e., action potentials) that propagate between LMCs through gap junctions, triggering the opening of voltage-gated Ca^2+^-channels [28] and entraining the contractions of LMCs [8,18]. Several intrinsic factors are known to regulate the contractile function of lymphatic vessels. These include the impairment of lymphatic contractions by forward-flow-induced wall shear stress, such that declines in contraction amplitude and frequency are observed experimentally in lymphatic segments subjected to various levels of intraluminal flow [29,30], as well as being predicted by numerical models [31]. Another important regulator is intraluminal pressure, where lymphatic vessels display higher frequency but lower amplitude contractions as intraluminal pressure rises [13,14,15].

Utilizing numerical modeling, we previously predicted that, although every LMC might have the capability of becoming a pacemaker, LMCs located at sites where current sink is biased in one direction, as opposed to both directions, are more likely to become the entraining cell, i.e., the dominant pacemaker [25]. Examples of these sites in the ex vivo scenario are LMCs located at the edges of the tissue (i.e., ends of the lymphatic segment), since electrical resistance is infinite in the direction of the cannulating pipette. In both in vivo and ex vivo scenarios, valve areas have been shown to have fewer and less-organized LMC-coverage and therefore, these are higher resistance sites with lower LMC-LMC coupling; LMCs at the edges of these valve areas have a bias towards conducting away from the valve, carrying more current as the signal spreads to neighboring LMCs [25].

Here, we adapted methods that we recently developed to assess the effects of differentially elevated downstream pressure on the initiation of lymphatic contractions, with the overall goal of determining if local increases in downstream pressure have any effect (perhaps negative) on lymphatic contraction wave entrainment. Understanding the regulation of pacemaking, specifically under differential pressure conditions, is important because it has been suggested that chronic elevated downstream pressure may be a factor that contributes to the development of lymphedema. Under these circumstances, the lymphatic system fails to cope with the overall adverse pressure gradient along the lymphatic vasculature that is exacerbated by gravitational forces.

We assessed the site of initiation and degree of propagation of lymphatic contractions in control mice, both in vivo and ex vivo. Interestingly, our results show that lymphatic contractions in vivo had a significantly higher probability to be initiated downstream in the lymphatic vasculature (i.e., somewhere near the popliteal lymph node). In vivo contractions propagated for at least 3 mm (the limit of the imaged field-of-view). Contractions of pressurized popliteal lymphatic vessels ex vivo displayed a similar result (i.e., higher probability for contractions to be initiated near the downstream pipette), although it was not statistically different (Figure 1 and Figure 2). Previous studies using bovine and rat mesenteric lymphatic vessels ex vivo, which although they did not assess the exact location of the pacemaking site, showed that propagation of lymphatic contractions could occur in both directions, with flow or against it [10,32]. The discrepancy between the consistent results observed in vivo and the non-statistically significant trend observed ex vivo is likely associated with the damage induced at the edges of lymphatic vessels in the ex vivo scenario, where vessels are cut and tied onto the cannulating pipettes. The current sink associated with cutting and tying a vessel at its ends may dominate over any other physiological current sink (e.g., LMCs near valve areas). In addition, the degree of vessel damage is likely different at each end and varies between vessels. In contrast, assessment of the contractile activity of lymphatic vessels in vivo was performed in the intact vasculature, where only physiological current sinks play a role [33].

Entrainment of the coordinated contraction of all LMCs along a lymphatic vessel requires strong electrical coupling, which we have previously shown to be mediated primarily via Cx45-containing gap junctions [7]. We have also demonstrated how important regional differences in electrical coupling may be for determining which LMC, or cluster of LMCs, will become the pacemaking site [25]. For this reason, pacemaking sites in isolated lymphatics are normally located at the ends of the preparation. Therefore, we used our smooth-muscle-specific Cx45-deficient mouse model, which displays impaired electrical coupling between LMCs, to determine 1) the effects of differentially elevated downstream pressure on lymphatic pacemaking and 2) the role that electrical coupling plays in determining the location of pacemaking sites, as well as determining which pacemaker dominates. First, we assessed the initiation of contractions in lymphatics from control (Cx45^fx/fx^ and WT) and Smmhc-CreER^T2^;Cx45^fx/fx^ mice pressurized to 2 cmH_2_O. Our results show that in control vessels, contractions were mainly initiated by a single dominant pacemaking site; however, over time most vessels displayed some contractions driven by a second, less-dominant initiation site. Cx45-deficient vessels, on the other hand, displayed significantly more pacemakers per unit length (Figure 5A); interestingly, while some pacemaking sites were more active than others, the differences in their activities were not statistically different (i.e., no dominant pacemaking sites could be determined). This is evident by the significantly higher switching probability in lymphatics from Smmhc-CreER^T2^;Cx45^fx/fx^ mice (Figure 5B). This is an important observation, because it suggests that critical to the development and establishment of a dominant pacemaker is the strong electrical coupling between all the LMCs in a lymphatic vessel. The higher number of pacemaking sites and the lack of dominant pacemakers in vessels with impaired electrical coupling between LMCs (i.e., Cx45-deficient vessels) result in an overall higher contractile frequency (Figure 5C), but not necessarily in more-efficient contractions as evident by the significantly impaired ejection fraction observed both ex vivo and in vivo (Figure 3C,F).

The absence of Cx45 from smooth muscle cells in blood vessels could potentially affect lymphatic contraction wave conduction, but that seems unlikely for at least two reasons: 1) no studies have shown electrical or chemical communication between arteries or veins and lymphatics (although two studies showed mechanical coupling if the vessels were in direct physical contact [34,35]). The separation between popliteal lymphatics and the saphenous vein in the mouse is at least 100–200 µm and the artery is even further away (several hundred microns), under a layer of muscle, so there is no physical coupling; thus, there is no direct interaction between popliteal lymphatics and either of these vessels; and 2) de Wit et al. [36] could not detect any functional deficits in conducted vasodilation or conducted vasoconstriction in arterioles of the cremaster microcirculation of Cx45 SM-KO mice (using Nestin-Cre to delete Cx45), nor were there any detectable differences in blood pressure in those mice, leaving open the question of the role for Cx45 in arteriolar function. If deletion of Cx45 from smooth muscle does not significantly affect arteriolar function, it is not likely to have an indirect effect on lymphatic function.

In the same set of lymphatic segments, we then asked the question whether imposing a differential pressure gradient, i.e., by elevating downstream pressure, would have any effect on the number of initiation sites, switching probability, and frequency. As previously mentioned, all these lymphatic segments had at least one valve, which closed and prevented any increase in upstream pressure as downstream pressure was differentially increased. The location of the closed valve is rather arbitrary as it depends on the structure and experimental preparation of each lymphatic vessel. Ideally, the closed valve would be located in the exact middle of the segment, so that the contributions from lower- and higher-pressure sides would be equal. In order to account for the location of the closed valve with respect to the segment midpoint, we calculated a normalized switching probability. Importantly, differential elevation of the downstream pressure caused an increase in the number of pacemaking sites, frequency, switching probability, and normalized switching probability in both control and Cx45-deficient vessels (Figure 8A–D and raw difference from control pressure in Figure 8E–H). Note that a selective increase in downstream pressure induced the development of new pacemaking sites even in control vessels. More importantly, while this increase in the number of active pacemakers (Figure 8A,E) also resulted in a higher switching probability (Figure 8C,D), control vessels continued to display a dominant pacemaker, i.e., mean raw and mean normalized switching probabilities < 20%, meaning one specific pacemaking continued to drive the majority of the contractions (Figure 8G,H). In contrast, in Cx45-deficient vessels, the adverse pressure gradient further increased the number of active pacemakers, the switching probability, and the contraction frequency.

Our data on connexin expression by FACS-sorted LMCs suggests that other connexin isoforms are normally present in the LMC layer and may also be involved in mediating electrical coupling between LMCs. Although multiple new pacemaker sites develop after Cx45 deletion, contraction waves continue to propagate to a limited extent, albeit at lower speeds and for shorter distances, which is evidence of residual LMC-LMC electrical coupling. It is possible, and perhaps likely, that other connexin isoforms, such as Cx43, Cx47, and Cx37, are upregulated in LMCs after Cx45 deletion (Figure 6). However, evidence for message does not always reflect protein expression and, because Western blotting is not feasible on the limited quantity of LMCs harvested from mouse lymphatics (nor would it specifically detect smooth muscle protein), the ultimate test of this idea is to assess whether contractile wave dysfunction is exacerbated in double knock-out mice, e.g., mice with smooth muscle deletion of Cx45 in combination with deletion of Cx43, Cx47, or Cx37. We are in the process of generating such mice for testing in future studies.

Cx45 SM-KO mice in this and our previous study do not develop peripheral lymphedema during the 10–12-month time course over which we have observed them [7]. Nor does it develop in mice expressing gain-of-function K_ATP_ channels in LMCs, which exhibit even greater lymphatic contractile dysfunction than shown here [24]. We hypothesize that both contractile impairment and a chronic gravitational load are likely needed to produce significantly, readily detectable peripheral lymphedema. Support for this idea was provided by Near Infrared Fluorescence (NIRF) imaging studies conducted under a gravitational load in the mouse hindlimb [7], which showed that lymph transport was significantly compromised in Cx45 SMC-KO mice only when they were imaged in a near-vertical body position but not in a horizontal position, whereas transport in WT mice was not impaired in either body position. The requirement for a gravitational load may be the same reason that lymphedema in humans is most readily apparent in dependent extremities and is often confined to the extremities. We suspect this is the explanation for the absence of overt peripheral lymphedema in many strains of mice with gene mutations known to cause lymphedema in humans [37,38,39] and predict that peripheral lymphedema would develop in those mice if they were subjected to a chronic gravitational load.

A final point worth discussing is the common misconception that increased contractile frequency always leads to a positive, improved outcome. As we have mentioned, in many parts of the body, the transport of lymph must be accomplished against an adverse pressure gradient; therefore, lymphatic contractions must generate a sufficient pressure head to open normally closed valves. An increase in contraction frequency normally comes at the expense of a decrease in contraction amplitude [15]; as frequency continues to increase, lymphatic contractions will eventually reach a limit where the generated propulsive pressure during a contraction is no longer sufficient to force open a closed valve subjected to an imposed adverse pressure gradient [16,17]; from this point on, pumping becomes negligible, as no fluid is being transported forward. Therefore, an increase in contractile frequency is beneficial only as long as contractions are still generating enough propulsive pressure to transport fluid across closed valves.

## 5. Conclusions

In conclusion, efficient lymph transport not only requires the strong, fully entrained contractions of all LMCs across multiple lymphangions, but also requires a dominant pacemaker for which high electrical coupling between LMCs through Cx45-containing gap-junctions is critical. In vessels from healthy controls, an increase in downstream pressure results in the recruitment/development of new pacemaking sites and increased contractile frequency while a dominant pacemaker is maintained. This may be a protective mechanism through which the lymphatic vasculature adapts to transient increases in downstream pressure and continues to transport fluid even against more demanding conditions. However, in patients presenting with chronic elevated downstream pressure, the pacemaking activity of lymphatics may be increased to such a degree that the high-frequency contractions do not generate sufficient propulsive pressure to efficiently transport fluid against the chronic elevated adverse pressure gradient. If this were the case, potential therapeutic approaches could be designed to slow down the overactive lymphatic pacemaking machinery. Alternatively, lowering the transmural pressure difference (P_lumen_-P_interstium_) would lower the pacemaking frequency, and this may be part of the beneficial effect of manual compression [40], which likely generates transiently high interstitial pressures [41] that would both compress the vessel and inhibit pacemaking. However, to target such mechanisms, further study and understanding of the ionic mechanisms underlying lymphatic pacemaking, and the factors that regulate them, are necessary.

## Figures and Tables

**Table 1:** Primer sequences used in this study.

Accession No.	Protein	Strand	Sequence	Amplicon (bp)
NM_080454	Cx47	s	AGC TCT GCC TTG TGC ATC TC	216
		as	CGT GTT GCA GGT GAA CTT GG	
NM_175452	Cx47	s	GAG AGG ATC AGC ATC CAG CC	262
		as	CGT GTT GCA GGT GAA CTT GG	
NM_008122	Cx45	s	GGT AAC AGG AGT TCT GGT GAA	140
		as	TCG AAA GAC AAT CAG CAC AGT	
NM_010288	Cx43	s	TGA GAG CCC GAA CTC TCC TT	258
		as	AGG CAG ACT GTT CAT CAC CC	
NM_008121	Cx40	s	CCA GAG CCT GAA GAA GCC AA	143
NM_001271628		as	CCG ATG ACT GTG GAG TGC TT	200
NM_008120	Cx37	s	GCT GCG CGC TAT TTA AGG C	131
		as	CAT GTT TCC AGG GCC TCT CT	
NM_001302497	Cx32	s	CAT GAG ACC ATA GGG GAG CTG	291
		as	ACG TGG GAG ATG GGG AAA AA	
NM_178596	Cx30.2	s	CGT CAT CTA CTC CAT GCA CCA	316
		as	GAC GGC GAA GTA GAA GAC CAC	
NM_001010937	Cx30	s	GAT CCC AAC GAG TGC CCT AAT	430
		as	CTG GAC ATC AGC AGC GGT AG	
NM_008125	Cx26	s	CAT TTC GGA CCA ACC CAG GA	148
		as	TGC CCC AAT CCA TCT TGT CC	
NM_008937	Prox1	s	GTA AGA CAT CAC CGC GTG C	218
		as	TCA TGG TCA GGC ATC ACT GG	
NM_009868	VE-Cadherin	s	CTT CCT TAC TGC CCT CAT TGT	313
		as	CTG TTT CTC TCG GTC CAA GTT	
NM_008713	e-NOS	s	CTG CCA CCT GAT CCT AAC TTG	143
		as	CAG CCA AAC ACC AAA GTC ATG	
NM_007392	α-Actin	s	GAG CTA CGA ACT GCC TGA C	129
		as	CTG TTA TAG GTG GTT TCG TGG A	
NM_013607	Myh11	s	AAG CTG CGG CTA GAG GTC A	238
		as	CCC TCC CTT TGA TGG CTG AG	
